# Safety of SGLT2 Inhibitors and Urinary Tract Infections in Clinical Practice—A Cross-Sectional Study

**DOI:** 10.3390/medicina60121974

**Published:** 2024-12-01

**Authors:** Liana Iordan, Vlad Florian Avram, Bogdan Timar, Adrian Sturza, Simona Popescu, Oana Albai, Romulus Zorin Timar

**Affiliations:** 1 Doctoral School of Medicine and Pharmacy, “Victor Babes” University of Medicine and Pharmacy, 300041 Timisoara, Romania; liana.iordan@umft.ro (L.I.); bogdan.timar@umft.ro (B.T.); timar.romulus@umft.ro (R.Z.T.); 2 Centre for Molecular Research in Nephrology and Vascular Disease, “Victor Babes” University of Medicine and Pharmacy, 300041 Timisoara, Romania; sturza.adrian@umft.ro (A.S.); popescu.simona@umft.ro (S.P.); albai.oana@umft.ro (O.A.); 3 Department of Diabetes, “Pius Brinzeu” Emergency County Hospital, 300723 Timisoara, Romania; 4 Second Department of Internal Medicine, “Victor Babes” University of Medicine and Pharmacy, 300041 Timisoara, Romania; 5 Department of Functional Sciences-Pathophysiology, Centre for Translational Research and Systems Medicine, “Victor Babes” University of Medicine and Pharmacy, 300041 Timisoara, Romania

**Keywords:** type 2 diabetes mellitus, diabetes medications, SGLT2 inhibitors, urinary tract infection, risk factors

## Abstract

*Background and Objectives*: Type 2 diabetes (T2DM) affects millions across the globe, generating a veritable public health issue through quality-of-life-reducing chronic complications, among which urinary tract infections are the most common. A shift in the disease managing paradigm from a glucose-centered view to a concept of cardio-reno-metabolic health has uniquely placed SGLT2 inhibitors as viable medication for the complex management of T2DM and its comorbidities. Some concerns have been raised over the increased likelihood of urinary tract infections (UTIs) associated with SGLT2 inhibitor use. The current study aims to evaluate the risk of developing urinary tract infections if patients with type 2 diabetes take SGLT2 inhibitors and determine those factors which make these patients more prone to develop this undesired complication. *Materials and Methods*: A cross-sectional, noninterventional evaluation of 328 patients with type 2 diabetes consecutively admitted to the Diabetes Clinic of “Pius Brinzeu” County Emergency Hospital in Timisoara, between January and February of 2024, was performed by examining medical charts and running statistical analyses using MedCalc version 22.26.0.0. *Results*: There was no statistical difference between patients taking SGLT2 inhibitors and those taking other glucose lowering medications when examining the presence of UTIs. Those patients with a higher HbA1c or BMI showed an increased predisposition to contracting UTI. The female gender was also associated with an increased likelihood of UTI. A further evaluation of the sublot of patients taking SGLT2 inhibitors revealed that not only higher BMI or HbA1c could be a predictor for the likelihood of developing UTI, but also a longer duration of T2DM was a predisposing factor. *Conclusions*: The use of SGLT2 inhibitors did not increase the likelihood of developing a urinary tract infection in this patient population.

## 1. Introduction

Type 2 diabetes mellitus (T2DM) is a chronic metabolic disease that affects millions of people across the globe, generating a veritable public health issue, as the ever-growing number of patients affected by this disease and its complications are presented with a lower quality of life [1,2]. Urinary tract infections (UTIs) are some of the most common complications seen in the care of patients with type 2 diabetes, while also representing the most common type of outpatient infection [3]. Impaired glucose control increases the risk of UTI as higher urine glucose levels promote the growth of pathogenic bacteria [4].

For many years the treatment of diabetes has focused primarily on reducing blood glucose and HbA1c as the main factors in managing T2DM and reducing the burden of long-term complications; however, in recent years, patient-centered views focusing on cardiovascular and renal risk factors have taken precedence, leading physicians to choose newer glucose-lowering agents in patient disease management [5]. The literature regarding the pathophysiology of T2DM has become more complex in recent decades, providing a larger range of therapeutic targets. SGLT2 inhibitors have been shown to be of great importance in the management of T2DM [6,7] and some of its most debilitating complications and comorbidities [8,9].

While SGLT2 inhibitors are an effective glucose-lowering agent and have substantial benefits in both the treatment of heart failure and chronic kidney disease [10,11,12], a fear of urinary tract infections and the associated impairment of glucose control have somewhat limited their prescription [13].

An important question for most physicians, after a new class of therapeutic agents becomes available for use in day-to-day practice, is how relevant the data from the clinical trials to the real-life clinical setting really is [14]. While cardiovascular safety is incontestable, issues have been raised regarding the non-cardiovascular safety of this therapeutic class [15].

The current work aims to clarify whether urinary tract infections are significantly associated with the use of SGLT2 inhibitors in real-life clinical practice and if so, identify the characteristics that made those patients susceptible to this side effect in an endeavor to improve long-term patient care.

## 2. Materials and Methods

A cross-sectional, noninterventional study was performed in a population of 328 patients with type 2 diabetes mellitus. Enrolment was based on a consecutive case principle. All patients with type 2 diabetes mellitus who provided consent and were admitted to the Diabetes Clinic of “Pius Brinzeu” County Emergency Hospital in Timisoara between January and February of 2024 were enrolled in the study.

All patients signed written consent forms prior to data collection and examination. Patients that could not provide informed consent or those who were unable to provide accurate anamnestic data were excluded from the study.

The study was approved by the local Ethics Committee of the “Pius Brinzeu” Timisoara Emergency Hospital and was conducted according to the principles stated in the Declaration of Helsinki.

Data collection was conducted from patient medical charts after taking the patients’ medical history and conducting a thorough anamnesis and physical exam by a doctor on admission to the clinic.

Data collected by physical examination and anamnesis included the following information: age, gender, current medication for the treatment of diabetes (dosage and duration of treatment), height, weight, body mass index (BMI), and symptoms.

Data collected from the patients’ medical charts included the following information: duration of diabetes, HbA1c, estimated glomerular filtration rate eGFR (using KGD-EPI creatinine equation 2021), urinary albumin creatinine ratio (UACR), urine culture, and urinalysis.

The diagnosis of UTI was achieved by examining the patient, cataloging symptoms, conducting urinalysis, and a positive urine culture, which was counted only once per patient. UTI was considered to be present when the patient presented both a urinalysis exam with more than 5 leucocytes per field and a urine culture with more than 10^5^ units forming colonies/field, even in the absence of symptoms.

Statistics tests were performed using MedCalc version 22.26.0.0. MedCalc Software Ltd. Belgium. Statistical significance between groups was determined using a Mann–Whitney U-test (for numerical variables with non-parametric distribution), respectively, and a Chi-square test (for categorical variables). To evaluate the impact of SGLT2 inhibitors, logistic regression models and ROC curve analyses were employed. The threshold of statistical significance considered in the study was a *p*-value of under 0.05.

## 3. Results

The current study lot comprised 328 subjects, of whom 162 (49.4%) were male and 166 (50.6%) were female. Patients were evaluated for the presence of urinary tract infections during normal screening, with tests showing a prevalence of 17.38% (57 cases) for urinary tract infections within the study group.

### 3.1. Study Lot Characteristics

The general characteristics of the study group are outlined summarily in Table 1. The median age was 63 years, while the median duration of diabetes was 8 years. Glucose control within the group was evaluated by measuring HbA1c, presenting a median value of 7.6%.

The treatment with SGLT2 inhibitors had a prevalence of 48.78% within the group, as 160 patients were already on this medication at the time of admission. No statistical difference was found between patients taking SGLT2 inhibitors and those taking other glucose-lowering medications. These results are summarily presented in Table 2.

### 3.2. Characteristics of Patients with Urinary Tract Infections Within the Study Group

Upon analyzing the results of the urine cultures in conjunction with the other parameters evaluated in the study, a positive association between improper glucose control (seen with higher values of HbA1c) and the presence of UTI can be seen. Moreover, those patients presenting a higher BMI were also more likely to preset UTI, as can be seen in Table 2. No significance was found between the presence of UTI and age, or the duration of diabetes or that of the eGFR.

Pathogens were implicated in causing UTI within the study population. In most cases (47), the UTIs were caused by bacteria, the most prevalent being *Escherichia coli*. The bacterial causal pathogens of UTI were as follows: *Escherichia coli*—26 cases (of which 15 used SGLT2 inhibitors), *Enterococcus*—8 cases (of which 4 used SGLT2 inhibitors), *Streptococcus agalactiae*—7 cases (of which 2 used SGLT2 inhibitors), and *Klebsiella pneumoniae*—6 cases (of which 3 used SGLT2 inhibitors). The fungal causal pathogen of UTI was *Candida albicans* in 10 cases (of which 7 used SGLT2 inhibitors). It should be pointed out that four cases presented with two causal pathogens for the UTI. In three cases, the associated pathogens were *Escherichia coli* and *Candida albicans* (of which two used SGLT2 inhibitors) and in one instance *Enterococcus* and *Candida albicans* (the patient used SGLT2 inhibitors).

Fungal pathogens were indeed more common in those patients taking SGLT2 inhibitors (seven vs. three cases), with three of the four cases of the associated bacterial and fungal infection being in patients taking this medication.

### 3.3. Risk Factors for Urinary Tract Infections Within the Study Group

Following the line of data exposed in the previous subsection, we further evaluated the risk of contracting a urinary tract infection by determining the odds ratio for BMI, HbA1c, and the use of an SGLT2 inhibitor for the treatment of T2DM. Moreover, we also evaluated if gender had an impact on whether the patient contracted a UTI. These data are summarized in Table 3.

These data show that taking an SGLT2 inhibitor did not increase the risk of contracting a UTI, but rather, patient BMI, gender, and HbA1c had a greater import in this.

As a higher patient HbA1c or BMI presented a greater risk in contracting a UTI, a ROC curve analysis was performed on these parameters and expressed in Figure S1 (available in Supplementary Materials) in order to determine which patients should benefit from more aggressive screening.

The analysis showed that patients with an HbA1c higher than 7.3% were more likely to develop urinary tract infections, while in the case of BMIs, the cut-off was at a BMI higher than 29 kg/m^2^.

### 3.4. Characteristics of Patients Taking SGLT2 Inhibitors

A detailed analysis of the sublot of patients taking SGLT2 inhibitors was undertaken. Table 4 summarily evaluates the sublot characteristics depending on the presence or the absence of a UTI. The results were largely the same as in the analysis of the entire patient population, apart from the duration of diabetes, which in this case, presented statistical significance, showing that there is a link between the duration of disease and the likelihood of contracting a UTI when taking SGLT2 inhibitors.

Similar to the entire population of the study, the odds ratio for BMI, HbA1c, and female gender was evaluated for the sublot of patients taking SGLT2 inhibitors and presented comparable results. The duration of diabetes was added to the analyzed parameters and yielded an odds ratio of 1.1499 with statistical significance (*p* < 0.05). The results are summarized in Table 5.

Taking these data into account, an ROC curve analysis for patient HbA1c, BMI, and duration of diabetes was evaluated in the sublot taking SGLT2 inhibitors. This is expressed in Figure S2 (available in Supplementary Materials).

The analysis yielded similar results in the case of the first two parameters, as the cut-offs for HbA1c and BMI were once again 7.3% and 29 kg/m^2^, respectively. Moreover, a duration of diabetes of more than 7 years was associated with a higher likelihood of developing a UTI in this patient sublot.

## 4. Discussion

The current study evaluated a group of patients with T2DM treated with various antidiabetic drugs including SGLT2 inhibitors. The main aim was to evaluate whether the use of SGLT2 inhibitors affected the risk of developing a UTI. The presence of T2DM itself can increase the risk of developing a UTI by as much as four times compared to patients with normal glucose control [16].

Table 4 shows that the use of an SGLT2 inhibitor for the treatment of T2DM does not increase the risk of contracting a UTI. This finding seems to be a matter of debate in the literature, as several studies have found conflicting results with some authors reporting an increased incidence of UTIs with the use of SGLT2 inhibitors [17,18]. Of note is that the concomitant use of sulfonylureas or insulin further increased the risk of UTIs [17]. On the other hand, there are also authors who reported that there was no significant increase in UTI incidence associated with the use of this medication as compared to other glucose-lowering agents [19,20,21]. It must be mentioned that even though certain studies report an increase in UTIs, the reports are consistent in showing that severe cases such as pyelonephritis and urosepsis are not as prevalent [16].

Regarding the pathogen responsible for UTIs, in Table 3, it is noticeable that *Escherichia coli* was the most common pathogen, as it is with most cases of UTI [3]. What is most noticeable in the current study is that the use of SGLT2 inhibitors did indeed favor fungal pathogens compared to other glucose-lowering drugs, a fact commonly reported in the literature [17,22]. This fact has also been confirmed by a systematic review and meta-analysis [15]. Improved hygiene in SGLT2-inhibitor-naïve patients could improve outcomes and diminish UTI incidence [23]. In a study conducted across multiple sites in India, it was shown that the incidence of UTI was also dependent on the duration of SGLT2 inhibitor use, with long-term users having a lower incidence of UTI caused by a bacterial pathogens [24]. In contrast, other authors have shown that the incidence of UTI increases within 6 months of treatment [17].

Regarding the predisposing factors of UTI, in this study, a higher BMI, higher HbA1c, and the female gender were shown to increase the risk of developing a UTI. The fact that more female patients developed UTI is not surprising as the particular anatomy of the female urinary tract and its proximity to the reproductive system are a well-known predisposing factor [3,18,25]. Regarding patient BMI, other authors have reported the same increase in the incidence of UTIs along with increased BMI, possibly due to difficult hygiene [25,26]. In contrast a sub-analysis by body mass index data analyzed from three clinical trials showed no link between BMI and UTIs [18].

In the case of HbA1c being linked to an increased incidence of UTI, it is most likely due to the fact that improper glucose control leads to increased glycosuria, which in turn provides adequate nourishments for the bacteria normally colonizing the genito-urinary tract [17,27]. However there are authors that suggest that a high HbA1c is not a risk factor for UTI when using SGLT2 inhibitors [28].

We have found that the duration of diabetes is linked to an increased probability of contracting a UTI in those patients already taking an SGLT2 inhibitor. The presence of T2DM is associated with a decrease in immunity and the body’s natural defense against infection; a longer duration of diabetes, especially with improper glucose control, is associated with more difficult glucose control and as such further weakens immunity [27,29].

It is worth mentioning that SGLT2 inhibitors provide many benefits, aside from lowering HbA1c, by improving cardiovascular and renal outcomes in patients with T2DM [12,30].

Regarding the above-mentioned points, one could draw the conclusion that SGLT2 inhibitors are generally safe concerning UTIs. It is worth mentioning that those patients with higher BMIs or with improper glucose control shown by a high HbA1c would benefit from a more diligent screening of UTI via urine culture after initiating treatment with SGLT2 inhibitors. We would suggest this to be done especially in female patients and those with a longer history of duration of T2DM. Moreover, given the benefits of SGLT2 inhibitors in both glucose control and general cardio-renal-metabolic health, their introduction into the patient care algorithm early on would provide better care and potentially diminish the risk of UTIs [5,9,10,11,19,20,27].

The limitations of the study would be the fact that it is a cross-sectional study and the limited number of variables taken into account when conducting the study, such as not considering the duration of SGLT-2 inhibitor use or personal hygiene aspects. We must also mention the fact that, for these reasons, the study was unable to determine a temporal relation between the stated risk factors and developing a UTI.

## 5. Conclusions

The treatment with SGLT2 inhibitors causes no more risk of urinary tract infections when compared to other glucose lowering medications, and as such is generally safe.

Greater attention must be shown to those patients with a longer duration of diabetes and higher values of HbA1c and BMI when screening for urinary tract infections, especially in the case of female patients.

## Figures and Tables

**Table 1:** Characteristics of patients treated with SGLT2 inhibitors vs. patients without SGLT2 inhibitors.

Variable	Taking SGLT2 Inhibitors		Not Taking SGLT2 Inhibitors		
	N	Median	n	Median	*p* *
Age	160	62	168	64	0.13
Duration of diabetes	160	8	168	9	0.95
BMI	160	28	168	27	0.14
HbA1c	160	7.60	168	7.6	0.24
eGFR	160	74	168	74	0.51
UACR	160	33	168	34	0.72

* Mann–Whitney test was performed between the two groups.

**Table 2:** Characteristics of patients with urinary tract infections vs. patients without urinary tract infections.

Variable	With Urinary Tract Infection		Without Urinary Tract Infection		
	N	Median	n	Median	*p* *
Age	57	64	270	62.5	0.24
Duration of diabetes	57	9	270	8	0.42
BMI	57	31	270	27	<0.0001
HbA1c	57	8.2	270	7.5	<0.0001
eGFR	57	68	270	74	0.06
UACR	57	49	270	32	<0.0001

* Mann-Whitney test was performed between the two groups.

**Table 3:** Risk factors for urinary tract infections.

Variable	Odds Ratio	95% CI	*p*
BMI	1.31	1.17 to 1.47	<0.0001
Female gender	2.70	1.46 to 5.01	0.0015
HbA1c	2.66	1.73 to 4.08	<0.0001
Taking an SGLT2 inhibitor	1.31	0.73 to 2.32	0.35

**Table 4:** Characteristics of patients taking SGLT2 inhibitors with urinary tract infections vs. patients taking SGLT2 inhibitors without urinary tract infections.

Variable	With Urinary Tract Infection		Without Urinary Tract Infection		
	N	Median	n	Median	*p* *
Age	31	66	129	62	0.06
Duration of diabetes	31	9	129	8	0.002
BMI	31	31	129	27	0.0009
HbA1c	31	7.9	129	7.5	0.0002
eGFR	31	68	129	74	0.14
UACR	31	51	129	31	<0.0001

* Mann–Whitney test was performed between the two groups.

**Table 5:** Risk factors for urinary tract infections for patients taking SGLT2 inhibitors.

Variable	Odds Ratio	95% CI	*p*
BMI	1.18	1.03 to 1.36	0.01
Female gender	2.57	1.07 to 6.19	0.03
HbA1c	2.47	1.40 to 4.34	0.001
Duration of diabetes	1.14	1.04 to 1.26	0.04

## Data Availability

The original contributions presented in the study are included in the article/supplementary material, further inquiries can be directed to the corresponding author.

## Supplementary Materials

The following supporting information can be downloaded at: https://www.mdpi.com/article/10.3390/medicina60121974/s1, Figure S1: ROC curves for HbA1c and BMI in the entire study lot.; Figure S2: ROC curves for HbA1c and BMI in the sublot taking SGLT2 inhibitors.
