# Supplementary figures and images for: Safety of SGLT2 Inhibitors and Urinary Tract Infections in Clinical Practice—A Cross-Sectional Study
Source: Medicina (Kaunas). 2024 Dec 1;60(12):1974. doi: 10.3390/medicina60121974 (PMC11678545; doi:10.3390/medicina60121974)

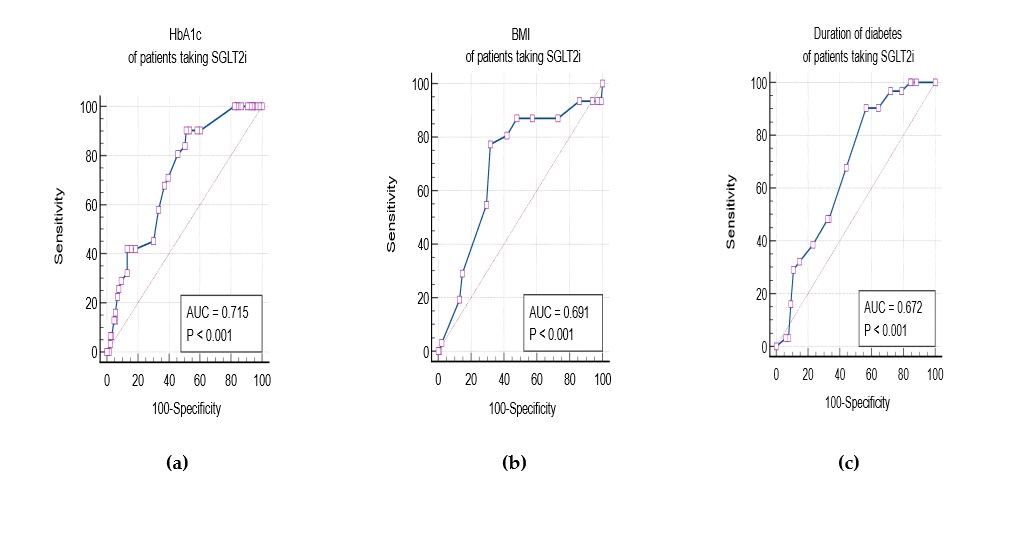

Supplement: Supplementary file 1 [file medicina-60-01974-s001.zip › medicina-3279425-supplementary/medicina-3279425-figures.jpg]

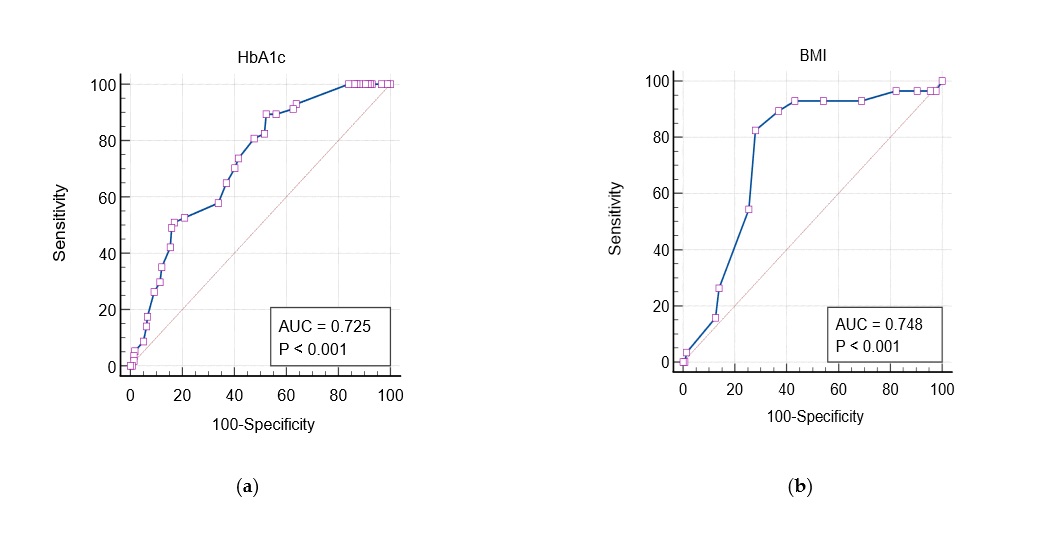

Supplement: Supplementary file 1 [file medicina-60-01974-s001.zip › medicina-3279425-supplementary/medicina-3279425-supplementary.jpg]
